# The interaction between *ASF1B* and *TLK1* promotes the malignant progression of low-grade glioma

**DOI:** 10.1080/07853890.2023.2169751

**Published:** 2023-03-22

**Authors:** Zifa Zhang, Shuming Liu

**Affiliations:** a Neurosurgery Department, Shanxi Bethune Hospital, Taiyuan, Shanxi, P. R. China; b Shanxi Academy of Medical Sciences, Taiyuan, Shanxi, P. R. China; c Emergency Department, Taiyuan People’s Hospital, Taiyuan, Shanxi, P. R. China

**Keywords:** ASF1B, TLK1, low-grade glioma, malignant progression

## Abstract

**Aim:**

Low-grade glioma (LGG), which is the second most frequent adult brain malignancy, severely threatens patients’ health and has a high recurrence rate. Histone H3/H4 chaperone anti-silencing function 1 B (*ASF1B*) has a tight association with the initiation and development of tumours. The expression and regulation mechanism of *ASF1B* in LGG were discussed.

**Methods:**

*ASF1B* expression in LGG patients as well as the association of *ASF1B* with overall survival and disease-free survival of LGG patients were predicted by GEPIA database. The independent prognostic value of ASF1B in LGG patients was investigated by TCGA database. RT-qPCR, together with western blot was applied for the assessment of ASF1B in LGG cell lines. After *ASF1B* expression was inhibited, CCK8 and colony formation assays judged cell proliferation. Flow cytometry analysis and TUNEL assay appraised cell cycle as well as apoptosis. Cell migratory and invasive capacities were measured by wound healing as well as Transwell assays. Western blot tested the expression of proliferation-, cycle-, apoptosis-, and metastasis-associated proteins. STRING and GeneMANIA database predicted the relationship between *ASF1B* and tousled-like kinase 1 (*TLK1)*. ChIP assay testified the affinity of *ASF1B* with *TLK1.* Subsequently, *TLK1* was overexpressed and *ASF1B* expression interfered, and the functional assays were executed.

**Results:**

*ASF1B* was discovered to be increased in LGG tissues and cells and indicates an unfavourable prognosis for LGG patients. *ASF1B* was not an independent prognostic factor for LGG. *ASF1B* deficiency obstructed the proliferation, cell cycle as well as metastasis of LGG cells, and induced cell death, which might be realized through the interaction with *TLK1*.

**Conclusion:**

The interaction between *ASF1B* and *TLK1* promoted the malignant progression of LGG.

**Key messages**
- TLK1 interacts with ASF1B.
- Interference with ASF1B inhibits the proliferative, invasive and migratory capabilities and induces the cycle arrest, along with the apoptosis of LGG cells.
- The interaction between ASF1B and TLK1 promotes the malignant progression of LGG.

## Introduction

Gliomas can be sorted into four grades in light of their malignancy [1]. Gliomas at grade II and grade III are known as lower-grade gliomas (LGG) [2]. LGG, which is a kind of adult brain tumour, is more common in young adults than in older people. Early manifestations of LGG patients include headache, vomiting, vision changes and increased intracranial pressure with the rapid growth of tumours, etc [3]. In addition, LGG has a high recurrence rate and poses a great threat to LGG patients’ health. Currently, LGG is treated with surgery combined with chemotherapy and radiotherapy. However, surgery can only achieve gross resection, and many glioma cells infiltrate into the normal brain tissue, making total tumour removal impossible [3]. Radiotherapy side effects, chemotherapy toxicity, “multi-drug resistance” and other problems remain challenging in LGG treatment [4]. Targeted gene therapy of LGG is the most exciting research area. Therefore, the search for biomarkers for LGG and the exploration of the mechanism of this disease is of tremendous significance to the diagnosis and treatment of LGG.

Histone H3/H4 chaperone anti-silencing function 1 (*ASF1*) participates in DNA-related processes together with transcriptional regulation [5]. *ASF1* is composed of two subtypes, including *ASF1A* and *ASF1B. ASF1B* is closely implicated in the initiation and development of numerous tumours through regulating cell proliferation [6]. A previous study has illuminated that enhancive ASF1B expression has a relation with the poor prognosis of lung adenocarcinoma [7]. *ASF1B* promoted the advancement of cervical cancer *via* stabilizing CDK9 [8]. In renal clear cell carcinoma, PKMYT1 was up-regulated and PKMYT1 was positively correlated with the anti-silencing effects of *ASF1B* gene [9]. *ASF1B* motivated the AKT signalling to facilitate cell proliferation and migration in renal clear cell carcinoma [10]. ASF1B suppression enhanced the apoptotic ability of prostate cancer cells by inactivating PI3K/Akt pathway [11]. In addition, gene expression profiles showed a significant increase in ASF1B expression following traumatic brain injury [12]. However, the influences of *ASF1B* on LGG have not been uncovered.

STRING and GeneMANIA databases predicted the possible interaction between *ASF1B* and tousled-like kinase 1*(TLK1)*. Being a serine/threonine protein kinase, *TLK1* regulated DNA replication as well as DNA repair pathways. Previous literature has shown that the knockdown of *TLK1* inhibited the survival of glioblastoma polymorphic cells [13]. miR-16 depletion promoted tumour advancement by activating *TLK1* in oral squamous cell carcinoma [14]. *TLK1* was overexpressed in both cholangiocarcinoma and cholangiocarcinoma cells and silencing of *TLK1* enhanced cisplatin-stimulated DNA damage [15].

Therefore, it is reasonable to hypothesize that the interaction between *ASF1B* and *TLK1* in LGG may affect the malignant progression of LGG. This paper is intended to discuss the role and regulatory mechanism of *ASF1B* in LGG.

## Materials and methods

### Database

GEPIA database (http://gepia.cancer-pku.cn) predicted *ASF1B* expression in LGG tissues as well as its correlation with overall survival or disease-free survival of LGG patients [16]. STRING (https://www.string-db.org/) [17] and GeneMANIA (http://genemania.org/) databases predicted the correlation between *ASF1B* and *TLK1* [18].

### Cell culture

LGG cell lines Res186, Res259, and Hs683 cells were supplied by the Shanghai Institute of Cell Biology and Chinese Academy of Sciences (Shanghai, China). Normal human astrocytes (NHA) were provided by Shanghai Hongshun Biotechnology Co., Ltd (Shanghai, China). All cells were cultivated in DMEM medium (Sigma) with 10% FBS (Sigma) as well as 1% antibiotics (Sigma) which were placed in a humidified atmosphere at 37 °C containing 5% CO_2_. The culture medium was replaced every 2 days.

### RT-qPCR

RNA was isolated *via* Trizol reagent (Guangzhou Shuoheng Biotechnology Co., Ltd, Guangzhou, China) in light of the standard protocol. With the application of Brilliant II Fast SYBR green QPCR master mix (Agilent Technologies, Santa Clara, CA, USA), reverse transcriptase reactions were operated. RT-qPCR was implemented with SYBR Green Master Mix (Beijing Baiao Laibo Technology Co., Ltd.) in 7500 FAST Real-Time PCR System (Bio-Rad Co., USA). Comparative Ct method was employed for the calculation of mRNA expression [19]. The following were the primer sequences: *ASF1B* forward: 5′-GCAGGATGCTGAGGAACCAT-3′, reverse: 5′-TTCAGGGTCCCAGTTGCTTC-3′; *TLK1* forward: 5′-ACTGGAAGTACGGGCAGTTG-3′, reverse: 5′- CTGTGGGAGGTTTGCGTTTG-3′; GAPDH: forward: 5′-AATGGGCAGCCGTTAGGAAA-3′, reverse 5′-GCGCCCAATACGACCAAATC-3′.

### Western blot

Proteins were isolated by RIPA lysis buffer (Shanghai Zeye Biotechnology Co., Ltd.). After the subjection to 10% SDS-PAGE, the transferring of proteins to PVDF membranes was operated. Subsequently, the membranes which were impeded with 5% skim milk was cultivated with primary antibodies overnight at 4 °C, following which was the cultivation with secondary antibody conjugated with the horseradish peroxidase for 2 h. Bands were then imaged by an enhanced chemiluminescent (ECL) kit (Bio-Rad Laboratories, Inc, CA, USA). GADPH served as a control to standardize the results. Protein bands were analysed by ImageJ software.

### Cell transfection

Short hairpin RNA *ASF1B* (sh-*ASF1B*) and its empty vector (sh-NC), and *TLK1*-specific pcDNA overexpression vector (Ov-*TLK1*) and its empty vector (Ov-NC) were purchased from Sangon Biotech (Shanghai, China). After cells (1 × 10^4^cells/well) that inoculated into 6-well plates reached 70% confluence, transduction of Ov-NC (10 µl/ml) and Ov-*TLK1* (10 µl/ml) or sh-*ASF1B* (10 µl/ml) and sh-NC (10 µl/ml) was implemented utilizing Lipofectamine 2000 (Shenzhen Ziker Biotechnology Co., Ltd, Shenzhen, China) [20]. Cells were collected following 48 h of transfection. Transduction efficacy was examined with RT-qPCR as well as western blot.

### Cell counting kit-8

Cell Counting kit-8 **(**CCK-8, MedChem Express LLC.) was adopted for the evaluation of cell viability in light of the instruction. After the supplementation of CCK-8 solution (10 μl), cells were incubated for 4 h. A microplate reader (Promega, WI, USA) was to analyse the OD value at 450 nm. After that, cell viability was evaluated with GraphPad Prism 7.0.

### Colony formation assay

Following corresponding induction, Res186 cells that were plated in 10-cm plates were stained with 0.05% crystal violet solution for imaging as well as colony counting 14 days later.

### Flow cytometry

Flow cytometry was employed for the appraisement of the cell cycle in line with the manufacturer’s protocol. Following corresponding induction, 70% EtOH was to immobilize cells for 15 min before PBS washing. Cell pellets were suspended in 500 µl PI solution (BD Biosciences, CA) before 40 min of cultivation at 37 °C. For flow cytometry analysis, cells were pelleted with 500 µl PBS.

### Tunel

The treated cells were subjected to 10 min of fixation with 4% paraformaldehyde. *In situ* cell death detection kit (Shanghai Hengfei Biotechnology Co., Ltd, Shanghai, China) was applied for the assessment of cell apoptotic rate.

### Wound healing

Res186 cells that were placed into 6-well plates were cultivated overnight till 100% cell fusion was achieved. The initial culture medium was replaced with DMEM containing mitomycin for a further 12 h of cultivation. With the help of a pipette tip, wounds were formed, after which the images were obtained [21].

### Transwell

The capability of cells to invade was appraised by employing ECMatrix gel (Chemicon)-coated transwell inserts. The upper chamber was filled with 5 × 10^4^ cells which were collected in serum-starved DMEM. The bottom chamber was composed of medium with 10% FBS. 24 h later, residual cells in the upper chambers were discarded. After that, 0.5% crystal violet was added for 10 min of staining and 70% ethanol was utilized for immobilization. Furthermore, six fields that were selected at random were captured and observed (100× magnification).

### Chromatin immunoprecipitation

Based on the Chromatin immunoprecipitation **(**ChIP kit, Millipore) protocol, ChIP assay was executed. Following cross-linking with 1% formaldehyde, DNA and protein were isolated by SDS lysis buffer, and sheared *via* sonication. ASF1B antibody (Abcam) was used for immunoprecipitation. RT-qPCR was conducted after the purification of precipitated DNA [22].

### Statistical analysis

All data that manifested in the format of means ± SD got were analysed using SPSS 19. Student’s *t*-test, one-way ANOVA, along with Tukey’s post hoc test was adopted for the demonstration of comparison among groups. Using Cox proportional risk regression for analysis, the risk ratio (HR) and 95% confidence interval (95% CI) were calculated. Differences were considered to be significant at *p* value less than 0.05. Each experiment was independently repeated three times.

## Results

### *ASF1B* is highly expressed in LGG tissues and has an association with the poor prognosis of LGG patients

**Figure 1.**
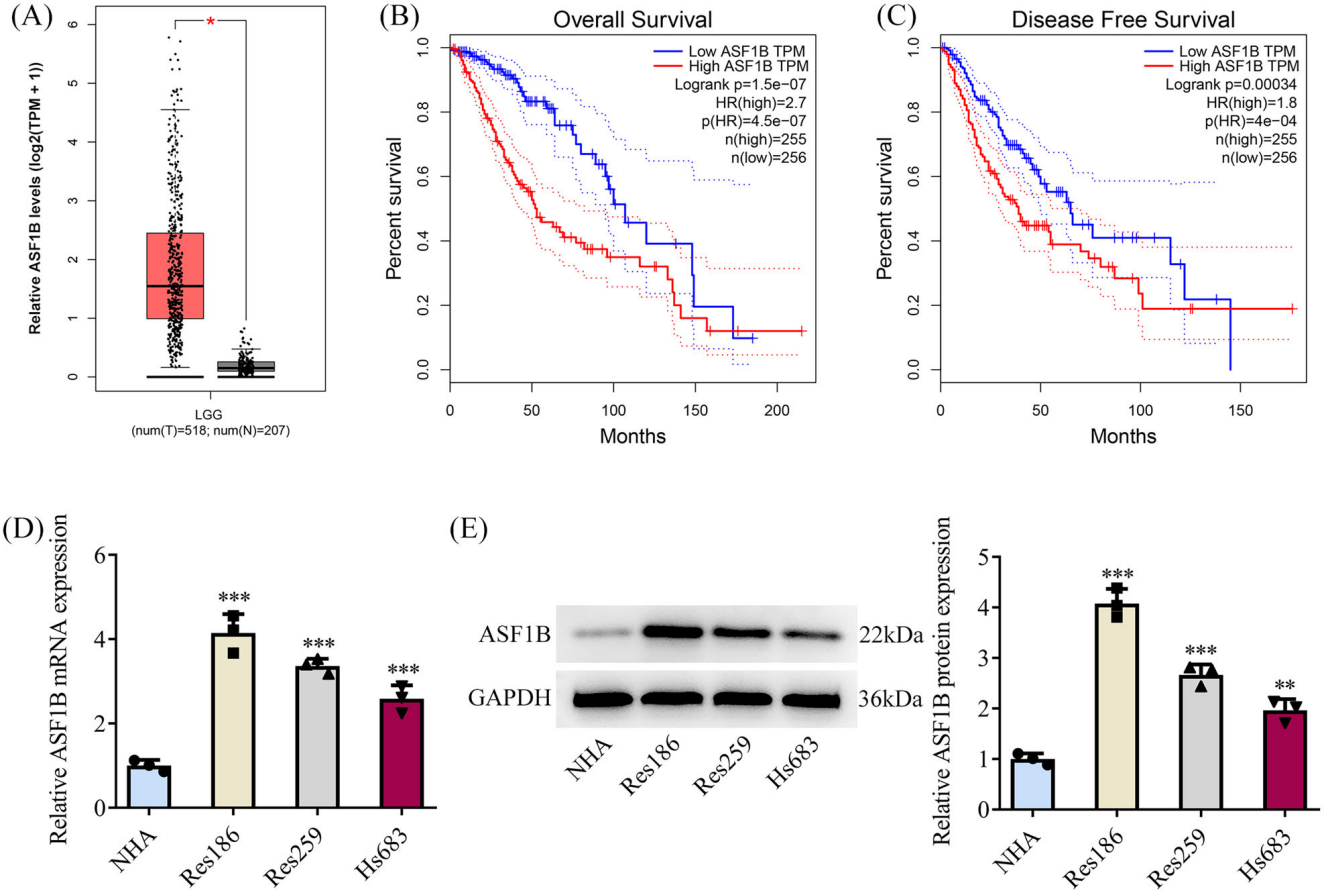
ASF1B is highly expressed in LGG tissues and is associated with poor prognosis of LGG patients. (A) GEPIA database showed that ASF1B was highly expressed in LGG patients. (B) GEPIA database showed that high ASF1B expression was significantly associated with low overall survival rate in LGG patients. (C) GEPIA database showed that high ASF1B expression was significantly associated with low disease-free survival rate of LGG patients. (D and E) RT-qPCR and Western blot were used to detect the expression of ASF1B in LGG cell lines. **p < 0.01, ***p < 0.001 vs NHA.

GEPIA database showed that *ASF1B* had higher levels in patients suffering from LGG (Figure 1(A)). Moreover, *ASF1B* elevation was prominently concerned with the low overall survival rate of LGG patients (Figure 1(B)). Increased *ASF1B* expression was markedly related to low disease-free survival of LGG patients (Figure 1(C)). Thus, we performed univariate and multivariate Cox regression analysis using age, sex, new tumours developed after the initial treatment, tumour status, radiation therapy and ASF1B expression as variables. From Tables 1 and 2, ASF1B expression was significantly associated with patient survival, while it could not be an independent factor for the results of univariate and multivariate Cox regression analysis. However, due to the significant differential expression of ASF1B in tissues and its relationship with patient prognosis, we decided further explore whether ASF1B can influence the function of LGG cells. Western blot and RT-qPCR results showed that *ASF1B* expression was remarkably ascended in LGG cell lines by contrast with NHA (Figure 1(D and E)). Res186 cells were chosen for follow-up experiments as *ASF1B* had the highest level in Res186 cells.

**Table 1.** Logistic regression analysis of ASF1B expression (univariate Cox analysis).

Characteristics	HR	HR (95%CI)	*p*
Age	1.06	1.04–1.07	<0.001
Sex			
Female		Ref	
Male	1.08	0.76–1.54	0.675
New tumour event after initial treatment			
No		Ref	
Yes	3.37	2.15–5.30	<0.001
Person neoplasm cancer status			
Tumour free		Ref	
With tumour	7.90	3.68–16.98	<0.001
Radiation therapy			
No		Ref	
Yes	1.95	1.26–3.02	0.003
ASF1B	1.68	1.39–2.03	<0.001

Ref: Reference; HR: Hazard Ratio; CI: Confidence Interval.

**Table 2.** Logistic regression analysis of ASF1B expression (multivariate Cox analysis).

Characteristics	HR	(95%CI)	*p*
Age	1.05	1.04–1.07	<0.001
New tumour event after initial treatment			
No		Ref	
Yes	2.77	1.75–4.40	<0.001
Person neoplasm cancer status			
Tumour free		Ref	
With tumour	6.04	2.73–13.35	<0.001
Radiation therapy			
No		Ref	
Yes	1.17	0.72–1.90	0.536
ASF1B	1.56	1.25–1.95	<0.001

Ref: Reference; HR: Hazard Ratio; CI: Confidence Interval.

### *ASF1B* interference inhibits the proliferative capability of LGG cells

**Figure 2.**
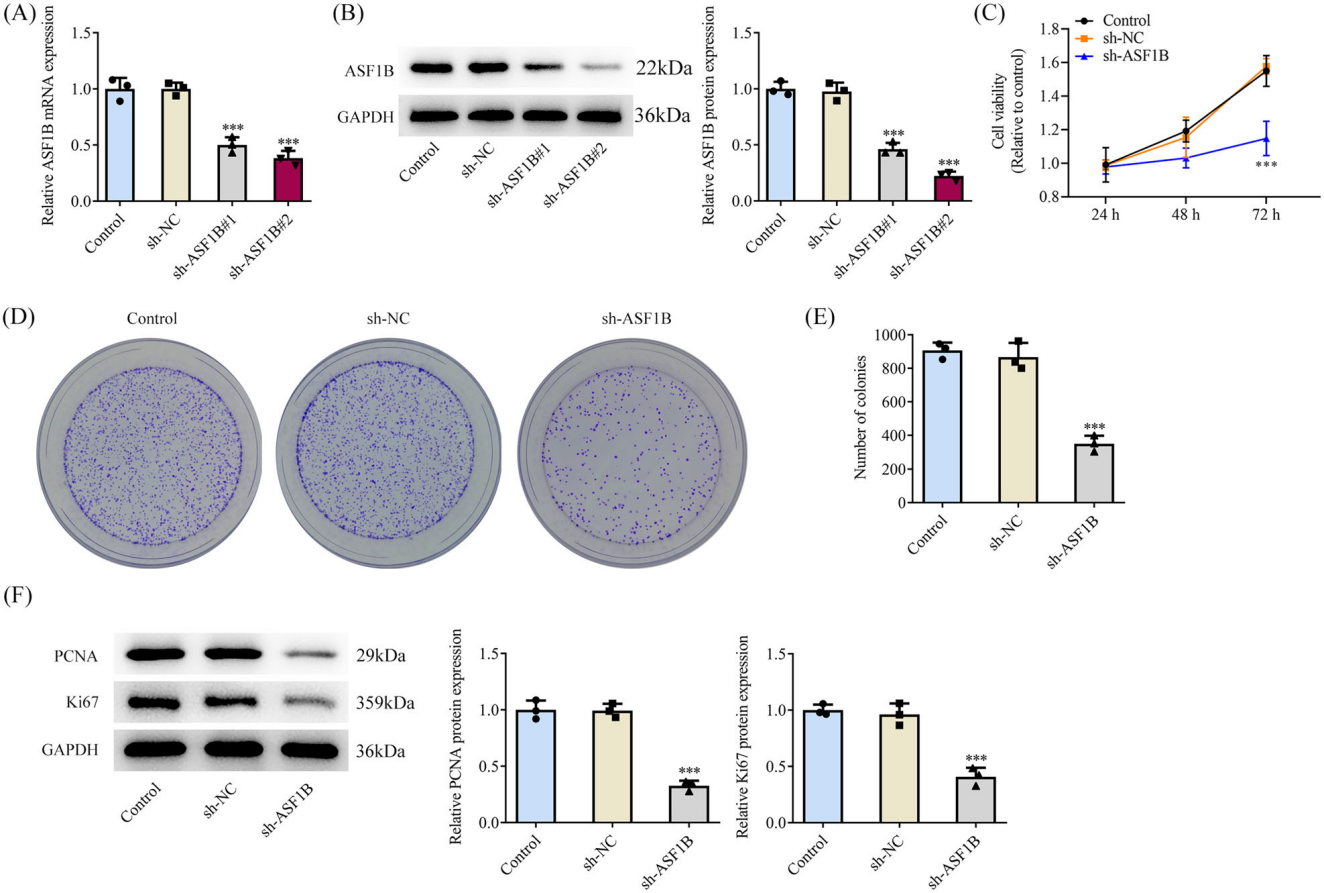
Interference with ASF1B inhibits proliferation of LGG cells. (A and B) RT-qPCR and Western blot were used to detect the expression of ASF1B after transfection. C.CCK-8 detected the cell viability. (D and E) Colony formation assay was used to detect the cell proliferation. (F) Western blot was used to detect the expression of PCNA and Ki67. ***p < 0.001 vs sh-NC.

*ASF1B* interference plasmid was constructed and its transfection efficiency was detected by RT-qPCR as well as Western blot (Figure 2(A and B)). Sh-*ASF1B*#2 remarkably diminished *ASF1B* expression, so sh-*ASF1B* #2 was chosen for follow-up experiments. CCK8 results corroborated that relative to sh-NC group, cell viability in sh-*ASF1B* group was distinctly declined (Figure 2(C)). Colony formation assay results exhibited that cell proliferative ability was noticeably weakened following *ASF1B* interference (Figure 2(D and E)). It was also manifested that PCNA and Ki67 expressions were cut down notably after *ASF1B* interference (Figure 2(F)).

### ASF1B interference induces cell cycle arrest and apoptosis in LGG

**Figure 3.**
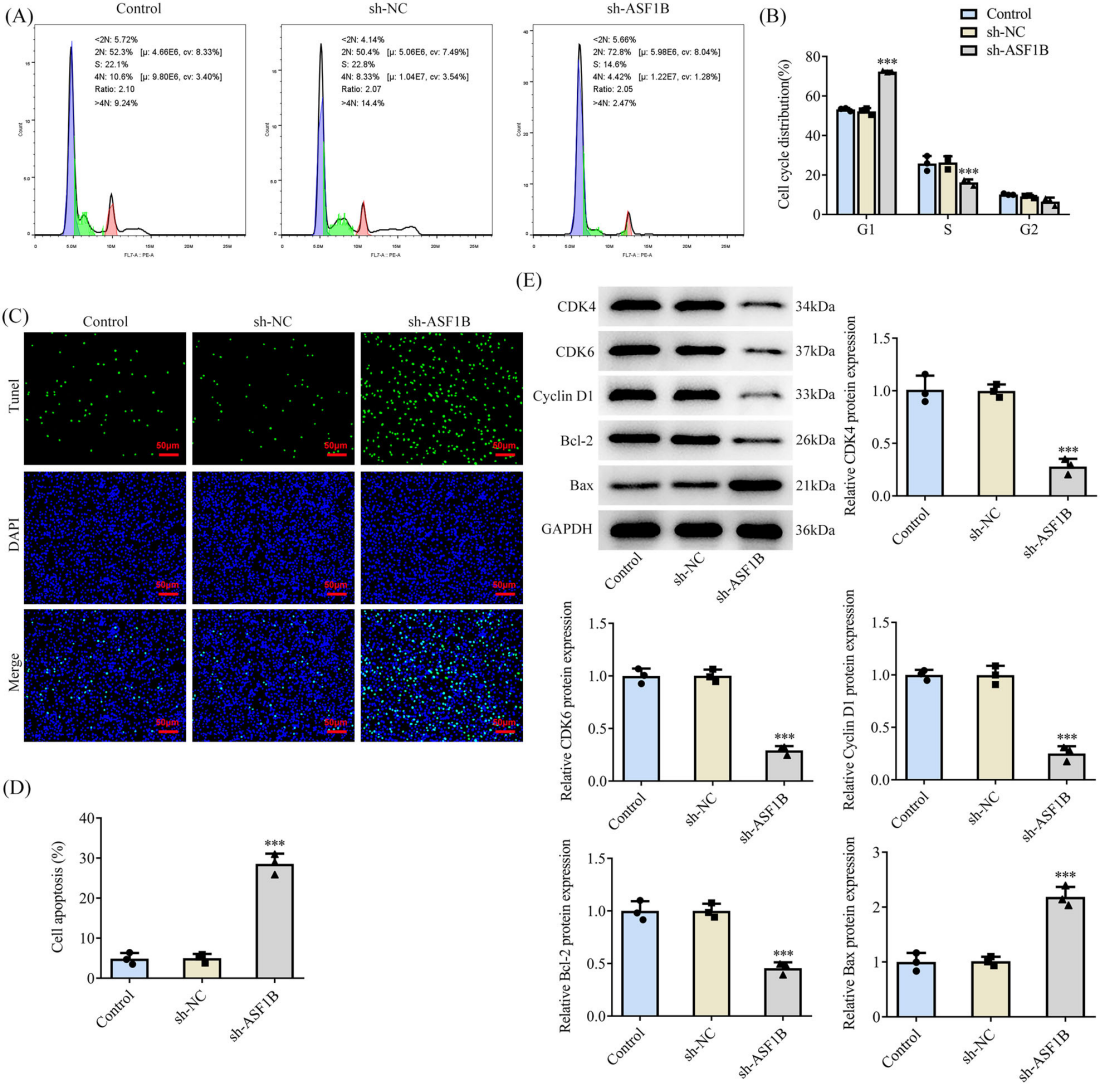
Interference with ASF1B induces cell cycle arrest and apoptosis in LGG. (A and B) Cell cycle was detected by flow cytometry. (C and D) TUNEL assay detected cell apoptosis. (E) Western blot was used to detect the expression of cell cycle and apoptosis-related proteins. ***p < 0.001 vs sh-NC.

Flow cytometry showed that cell cycle arrest occurred after *ASF1B* interference (Figure 3(A and B)). Relative to the sh-NC group, the apoptosis of sh-*ASF1B* group was dramatically strengthened by sh-*ASF1B* (Figure 3(C and D)). As Figure 3(E) depicted, the expression of cell cycle-related proteins CDK4, CDK6, Cyclin D1 and anti-apoptotic protein Bcl-2 in sh-*ASF1B* group were apparently lessened, while the expression of pro-apoptotic protein Bax expression was clearly aggrandized when compared to the sh-NC group.

### ASF1B interference inhibits the invasive and migratory capabilities of LGG cells

**Figure 4.**
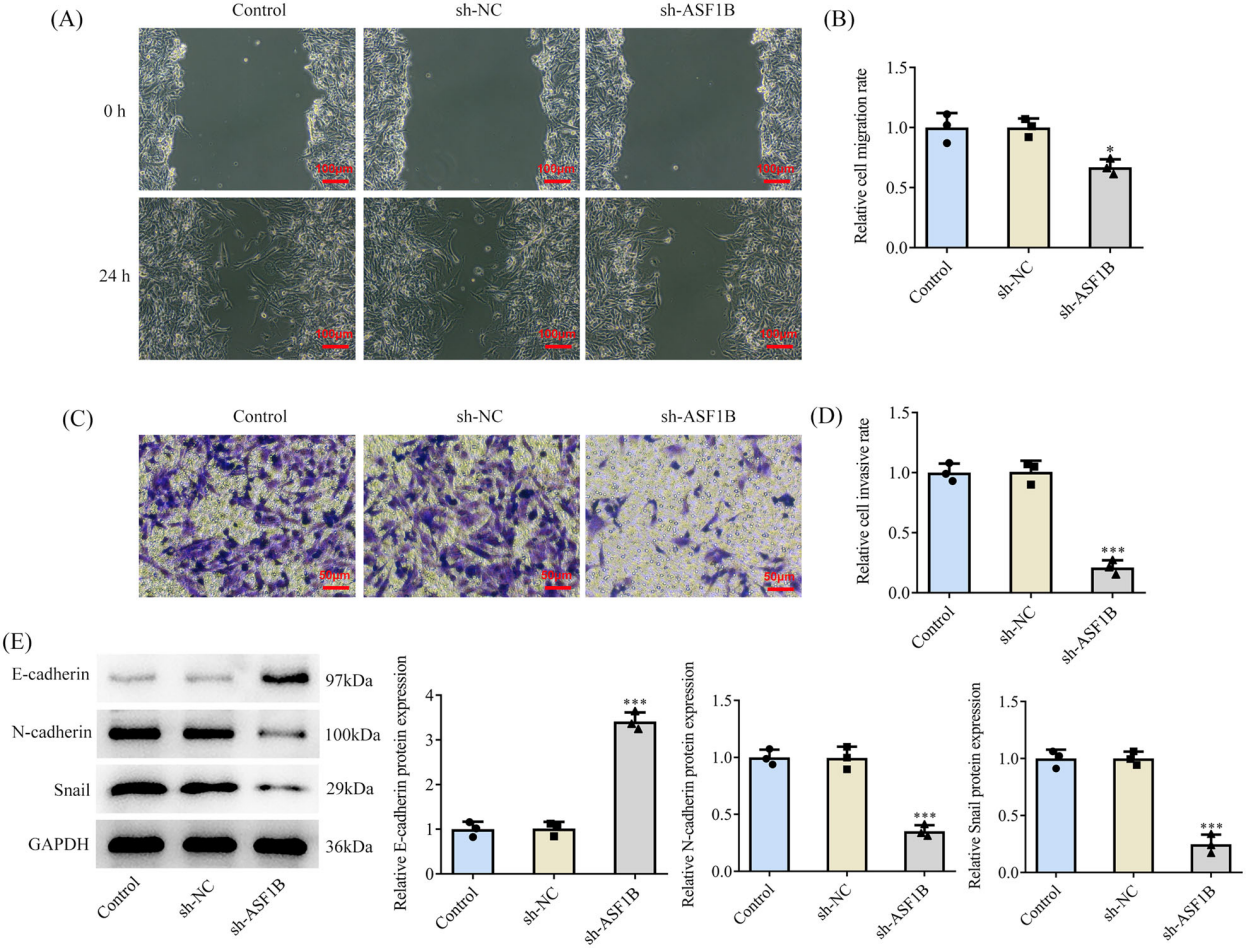
Interference with ASF1B inhibits invasion and migration of LGG cells. (A and B) Wound healing was used to detect the cell migration. (C and D) Transwell was used to detect the cell invasion. (E) Western blot was used to detect the expression of cell migration and invasion-related proteins. *p < 0.05, ***p < 0.001 vs sh-NC.

Results from wound healing (Figure 4(A and B)) and Transwell (Figure 4(C and D)) assays substantiated that cell invasive as well as migratory capacities in sh-ASF1B group were observably attenuated in contrast with the sh-NC group. It was also found that E-cadherin expression was ascended while N-cadherin and Snail expression were diminished after *ASF1B* interference (Figure 4(E)).

### *TLK1* interacts with *ASF1B*

**Figure 5.**
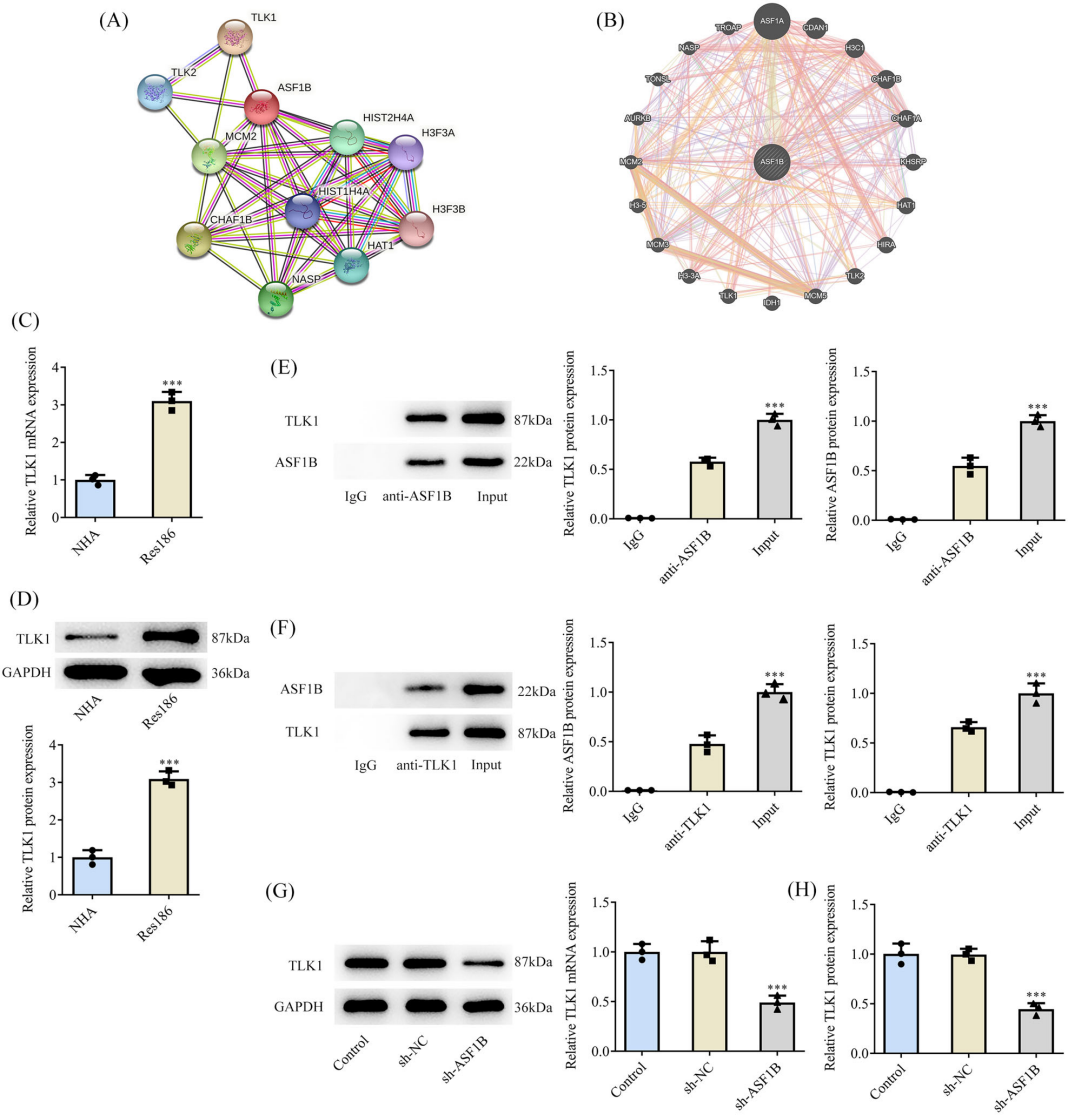
TLK1 interacts with ASF1B. STRING (A) and GeneMANIA (B) databases uncovered potential interactions between ASF1B and TLK1. (C and D) RT-qPCR and Western blot were used to detect the expression of TLK1 in LGG cells. ***p < 0.001 vs NHA. (E and F) The targeted binding of ASF1B and TLK1 was verified by ChIP assay. ***p < 0.001 vs IgG. (G and H) RT-qPCR and Western blot were used to detect the expression of TLK1 after depletion of ASF1B. ***p < 0.001 vs sh-NC.

STRING and GeneMANIA databases uncovered potential interactions between *ASF1B* and *TLK1* (Figure 5(A and B)). In addition, *TLK1* expression was noticeably fortified in Res186 cells (Figure 5(C and D)). The targeted binding of *ASF1B* and *TLK1* was verified by ChIP assay (Figure 5(E and F)). In addition, inhibition of *ASF1B* expression significantly reduced the expression of *TLK1* in Res186 cells (Figure 5(G and H)).

### Upregulation of *TLK1* partially reverses the effects of *ASF1B* interference on LGG cells

**Figure 6.**
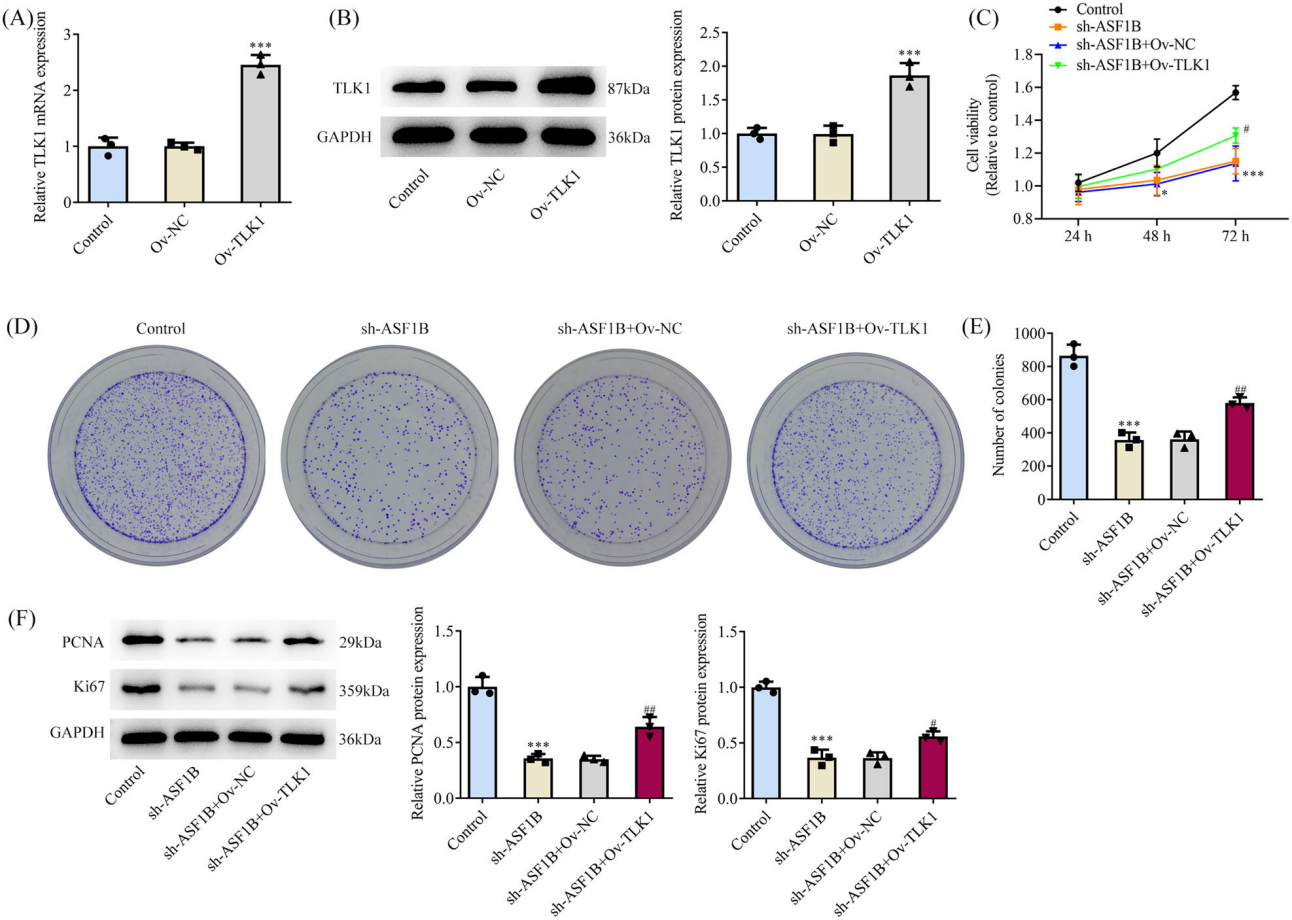
Upregulation of TLK1 partially reverses the effect of ASF1B interference on cell proliferation. **(**A and B) RT-qPCR and Western blot were used to detect the expression of TLK1 after transfection. ***p < 0.001 vs Ov-NC. (C) CCK-8 detected the cell viability. (D and E) Colony formation assay was used to detect the cell proliferation. F. Western blot was used to detect the expression of PCNA and Ki67. *p < 0.05, ***p < 0.001 vs Control; #*p* < 0.05, ##*p* < 0.01 vs sh-ASF1B + Ov-NC.

**Figure 7.**
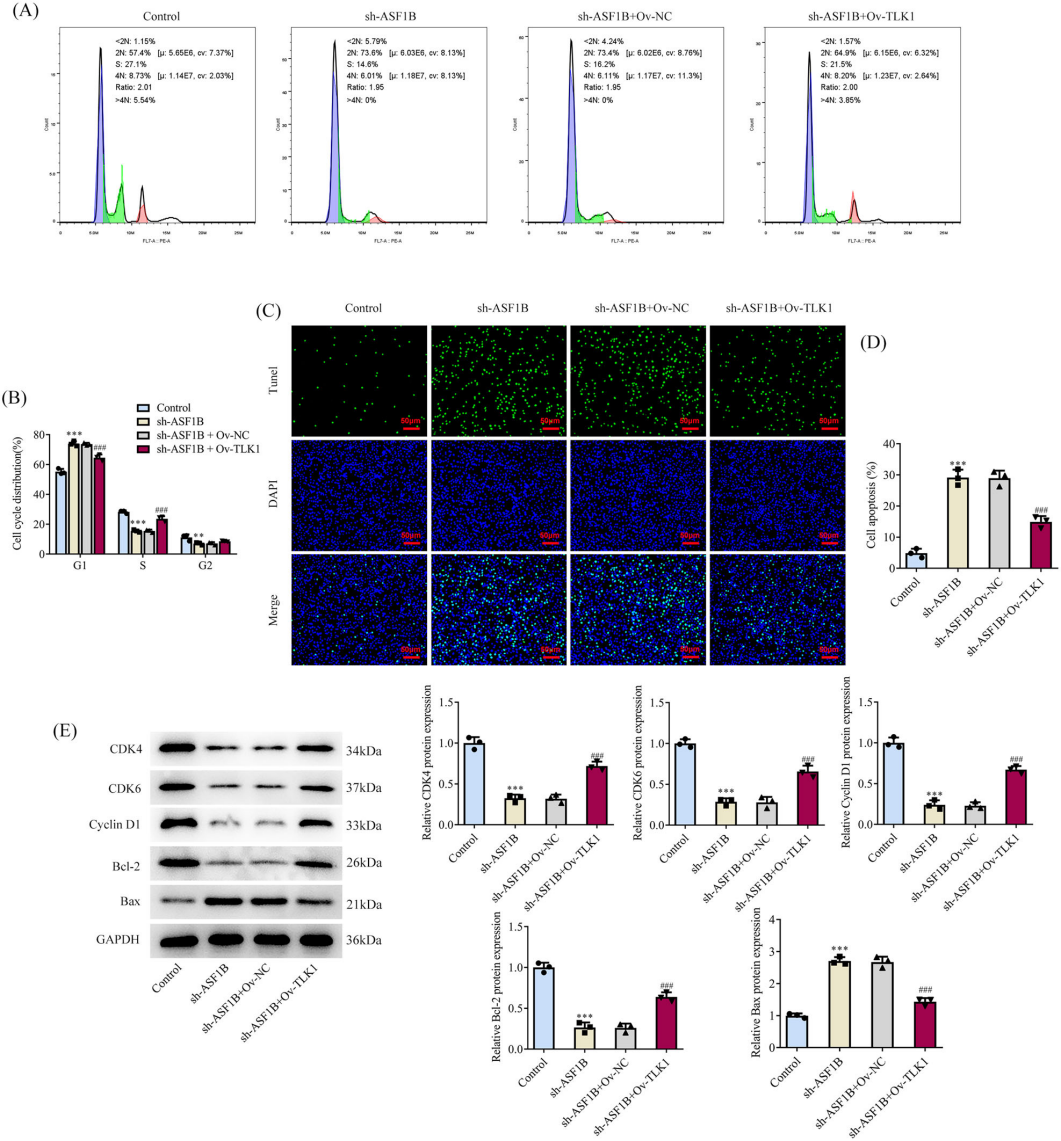
Upregulation of TLK1 partially reverses the effects of ASF1B interference on cell apoptosis. (A and B) Cell cycle was detected by flow cytometry. (C and D) TUNEL assay detected cell apoptosis. E and F. Western blot was used to detect the expression of cell cycle and apoptosis-related proteins. ***p < 0.001 vs Control; #*p* < 0.05, ###*p* < 0.001 vs sh-ASF1B + Ov-NC.

**Figure 8.**
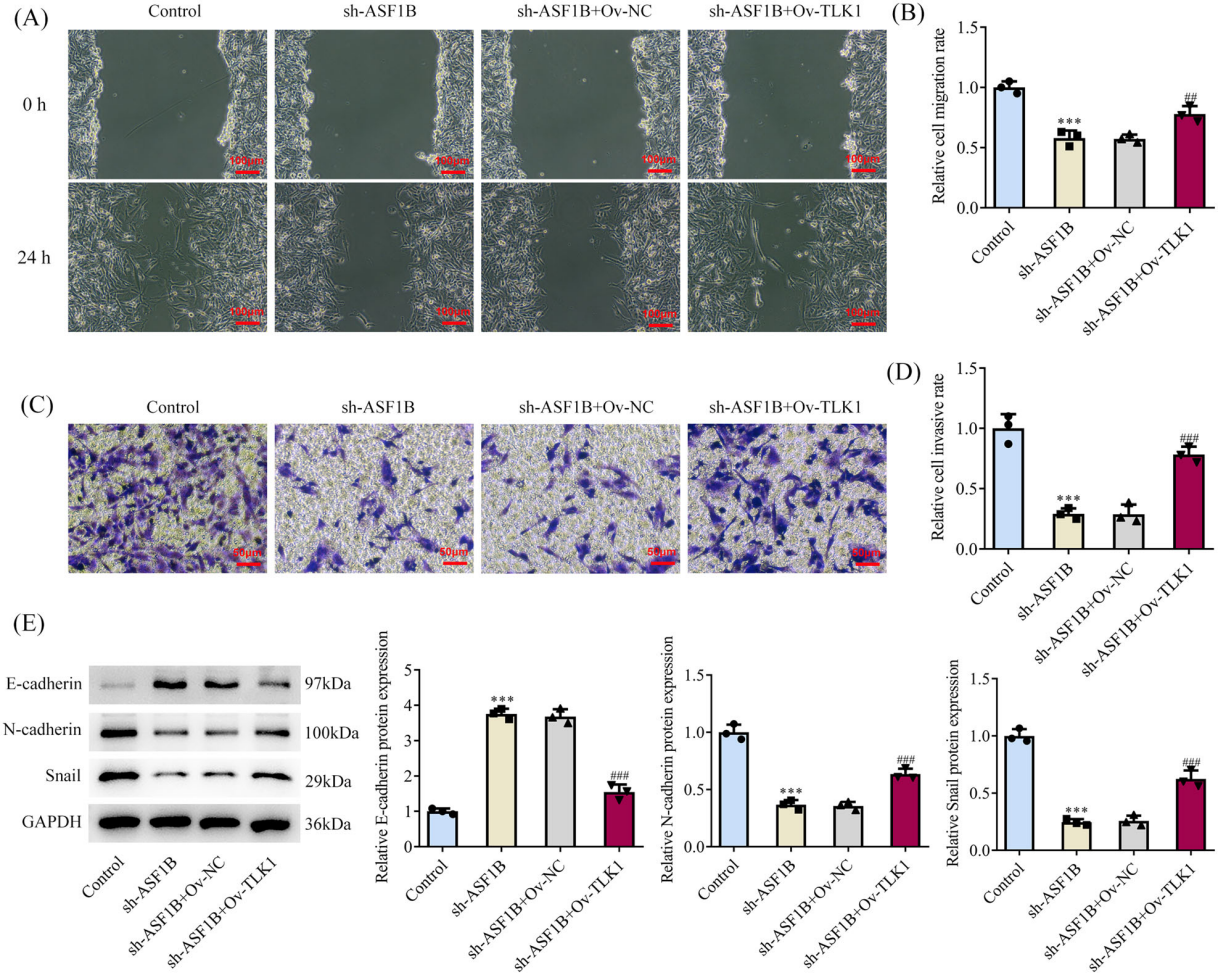
Upregulation of TLK1 partially reverses the effects of ASF1B interference on cell migration and invasion. (A and B) Wound healing was used to detect the cell migration. (C and D) Transwell was used to detect the cell invasion. (E) Western blot was used to detect the expression of migration and invasion-related proteins. ***p < 0.001 vs Control; ##*p* < 0.01, ###*p* < 0.001 vs sh-ASF1B + Ov-NC.

Next, the regulation mechanism of *ASF1B* in LGG was discussed and *TLK1* overexpression plasmid was constructed, RT-qPCR, and Western blot was applied to examine the transduction efficacy (Figure 6(A and B)). CCK8 results elaborated that relative to sh-*ASF1B* + Ov-NC group, cell viability in sh-*ASF1B* + Ov-*TLK1* group was evidently aggravated (Figure 6(C)). The proliferation ability of sh-*ASF1B* + Ov-*TLK1* group was markedly increased (Figure 6(D and E)), accompanied by aggrandized PCNA and Ki67 expression relative to sh-*ASF1B* + Ov-NC group (Figure 6(F)). Relative to sh-*ASF1B* + Ov-NC group, cell cycle arrest was reversed and apoptosis was conspicuously cut down in sh-*ASF1B* + Ov-*TLK1* group (Figure 7(A–D)). As Figure 7(E and F) demonstrated, the expression of CDK4, CDK6, Cyclin D1 and Bcl-2 were augmented in the sh-*ASF1B* + Ov-*TLK1* group, while Bax expression was reduced by contrast with the sh-*ASF1B* + Ov-NC group. Wound healing and Transwell results uncovered that cell migration (Figure 8(A and B)) and invasion (Figure 8(C and D)) were notably facilitated in sh-*ASF1B* + Ov-*TLK1* group relative to the sh-*ASF1B* + Ov-NC group. Western blot analysis of invasion and migration-related proteins exposed that E-cadherin expression in the sh-*ASF1B* + OV-*TLK1* group was cut down, while N-cadherin and Snail expression were fortified by contrast with the sh-*ASF1B* + Ov-NC group (Figure 8(E)).

## Discussion

Glioma is the most prevalent brain malignancy. LGG belongs to grade II-III glioma and is not as aggressive as high-grade glioma [23]. However, LGG is more aggressive and will inevitably develop into high-grade gliomas with the passage of time [24]. Recent studies on LGG have focused on molecular genetics, but the mechanisms of the migration, invasion, together with apoptosis in LGG remain elusive. Therefore, it is very important to further study the relevant mechanisms covering migration, invasion, and apoptosis in LGG.

Zhu et al. [25] found through The Cancer Genome Atlas database (TCGA), genotypic tissue expression (GTEx), and the Chinese Gliomas Genome Atlas database (CGGA) that the expression of *ASF1B* in glioma tissue was significantly higher than that in normal tissue, and the increased expression of *ASF1B* was associated with poor prognosis of glioma patients. In this study, through GEPIA database, *ASF1B* was noticed to possess elevated expression in LGG tissues, and enhancive *ASF1B* expression had noticeable relation with unfavorable overall survival and disease-free survival rate of LGG patients. Clearly, our results are consistent with those of Zhu et al. Subsequently, *ASF1B* expression was also prominently increased in LGG cells. A previous study showed that *ASF1B* is overexpressed in multiple cancers and is closely associated with cancer survival [26]. Next, it was found that interference with *ASF1B* in Res186 cells could remarkably halt cell proliferation, metastasis and stimulate cell cycle arrest and apoptosis in LGG in our study. *ASF1B* could enhance the migratory as well as invasive capabilities of lung cancer cells *via* the modulation of p53-mediated epithelial-mesenchymal transformation signalling pathway [27]. MiR-214 inhibited the proliferative ability and promoted the apoptotic rate of myeloma cells by down-regulating *ASF1B* [28]. *ASF1B* possessed fortified expression, and *ASF1B* absence could accelerate apoptosis, together with cell cycle arrest in hepatocellular carcinoma [29]. In addition, *ASF1B* was also a prognostic marker for breast cancer as well as lung adenocarcinoma, and occupied an important position in the initiation and progression of tumours [7,30]. However, the impacts of *ASF1B* and its regulatory mechanism in LGG have not been reported. Moreover, gene expression profiles showed a significant increase in ASF1B expression following cerebral trauma injury [12]. Therefore, our results, together with previously published studies, suggest that ASF1B acts as a cancer-promoting factor in the development of multiple cancers, providing a basis for the use of *ASF1B* as a therapeutic target for LGG or other cancers.

Next, the regulatory mechanism of *ASF1B* in LGG cells was deeply probed into. Potential interactions between *ASF1B* and *TLK1* were revealed through relevant databases. Subsequently, the transcriptional regulation of *TLK1* by *ASF1B* was confirmed through experiments in this study. It was confirmed that *TLK1* mainly functioned dependent on mediating the H3/H4 histone chaperone *ASF1*, and *ASF1B* is phosphorylated by TLKs during DNA replication on its C-terminal tail residues S169 and S198 [31]. TLK phosphorylation of ASF1 regulated its stability, while in vertebrates, TLK1-mediated phosphorylation of several sites on the C-terminal tail of ASF1 promoted its binding affinity for the histone H3/H4 heterodimer [32]. So in LGG, does ASF1B play a regulatory role in tumour by regulating TLK1? Our results showed that *ASF1B* and *TLK1* observably had a positive correlation in LGG patients and *TLK1* was discovered to be distinctly overexpressed in LGG cells. It was shown that *TLK1* expression was elevated in glioma tissues, and had correlation with large tumour volume and higher grade of glioma [33]. Our findings are consistent with those of others. Finally, we found that *TLK1* elevation partially reversed the impacts of *ASF1B* interference on cell viability, metastasis, cell cycle as well as apoptosis in LGG. The results of Ibrahim et al. [13] also showed that knockdown of *TLK1* inhibited the survival of glioblastoma polymorphic cells. Overexpression of *TLK1* can significantly promote the growth, migration, and invasion of glioma cells, and inhibit cell apoptosis [33]. Over all, this study helped us better understand the molecular mechanism by which ASF1B regulated LGG occurrence and development.

There are some disadvantages in this paper as well. First of all, only Res186 cell line was selected in our experiments and other LGG cell lines need to be chosen for validation in future experiments. Second, only *in vitro* cell experiments were carried out and animal experiments *in vivo* will be performed in the future to verify this finding.

## Conclusion

In conclusion, this paper confirms that the interaction between *ASF1B* and *TLK1* promotes the malignant progression of LGG, which lays a theoretical foundation for the discussion of targeted therapy for LGG.

## Data Availability

The analysed data sets generated during the present study are available from the corresponding author on reasonable request.

## References

[1] Ostrom QT, Bauchet L, Davis FG, et al. The epidemiology of glioma in adults: a “state of the science” review. Neuro Oncol. 2014;16(7):896–913. 24842956 10.1093/neuonc/nou087 PMC4057143

[2] Yang Y, Yan LF, Zhang X, et al. Glioma grading on conventional MR images: a deep learning study with transfer learning. Front Neurosci. 2018;12(804):804. 30498429 10.3389/fnins.2018.00804 PMC6250094

[3] Youssef G, Miller JJ. Lower grade gliomas. Curr Neurol Neurosci Rep. 2020;20(7):21. 32444979 10.1007/s11910-020-01040-8 PMC7244462

[4] McDuff SGR, Dietrich J, Atkins KM, et al. Radiation and chemotherapy for high-risk lower grade gliomas: choosing between temozolomide and PCV. Cancer Med. 2020;9(1):3–11. 31701682 10.1002/cam4.2686 PMC6943166

[5] Paul PK, Rabaglia ME, Wang CY, et al. Histone chaperone ASF1B promotes human beta-cell proliferation via recruitment of histone H3.3. Cell Cycle. 2016;15(23):3191–3202. 27753532 10.1080/15384101.2016.1241914 PMC5176155

[6] Abascal F, Corpet A, Gurard-Levin ZA, et al. Subfunctionalization via adaptive evolution influenced by genomic context: the case of histone chaperones ASF1a and ASF1b. Mol Biol Evol. 2013;30(8):1853–1866. 23645555 10.1093/molbev/mst086

[7] Feng Z, Zhang J, Zheng Y, et al. Elevated expression of ASF1B correlates with poor prognosis in human lung adenocarcinoma. Per Med. 2021;18(2):115–127. 33576264 10.2217/pme-2020-0112

[8] Liu X, Song J, Zhang Y, et al. ASF1B promotes cervical cancer progression through stabilization of CDK9. Cell Death Dis. 2020;11(8):705. 32848135 10.1038/s41419-020-02872-5 PMC7449975

[9] Chen P, Zhang Z, Chen X. Overexpression of PKMYT1 facilitates tumour development and is correlated with poor prognosis in clear cell renal cell carcinoma. Med Sci Monit. 2020;26:e926755. 33024069 10.12659/MSM.926755 PMC7549326

[10] Jiangqiao Z, Tao Q, Zhongbao C, et al. Anti-silencing function 1B histone chaperone promotes cell proliferation and migration via activation of the AKT pathway in clear cell renal cell carcinoma. Biochem Biophys Res Commun. 2019;511(1):165–172. 30777326 10.1016/j.bbrc.2019.02.060

[11] Han G, Zhang X, Liu P, et al. Knockdown of anti-silencing function 1B histone chaperone induces cell apoptosis via repressing PI3K/Akt pathway in prostate cancer. Int J Oncol. 2018;53(5):2056–2066. 30132513 10.3892/ijo.2018.4526 PMC6192734

[12] Zhao ZJ, Wei DP, Zheng RZ, et al. The gene coexpression analysis identifies functional modules dynamically changed after traumatic brain injury. Comput Math Methods Med. 2021;2021:5511598. 33953790 10.1155/2021/5511598 PMC8068551

[13] Ibrahim K, Abdul Murad NA, Harun R, et al. Knockdown of tousledlike kinase 1 inhibits survival of glioblastoma multiforme cells. Int J Mol Med. 2020;46(2):685–699. 32468002 10.3892/ijmm.2020.4619 PMC7307829

[14] Hu S, Wang H, Yan D, et al. Loss of miR-16 contributes to tumour progression by activation of tousled-like kinase 1 in oral squamous cell carcinoma. Cell Cycle. 2018;17(18):2284–2295. 30252587 10.1080/15384101.2018.1526601 PMC6226226

[15] Takayama Y, Kokuryo T, Yokoyama Y, et al. Silencing of tousled-like kinase 1 sensitizes cholangiocarcinoma cells to cisplatin-induced apoptosis. Cancer Lett. 2010;296(1):27–34. 20381954 10.1016/j.canlet.2010.03.011

[16] Li C, Tang Z, Zhang W, et al. GEPIA2021: integrating multiple deconvolution-based analysis into GEPIA. Nucleic Acids Res. 2021;49(W1):W242–W246. 34050758 10.1093/nar/gkab418 PMC8262695

[17] Szklarczyk D, Franceschini A, Wyder S, et al. String v10: protein-protein interaction networks, integrated over the tree of life. Nucleic Acids Res. 2015;43 issue):D447–452. 25352553 10.1093/nar/gku1003 PMC4383874

[18] Warde-Farley D, Donaldson SL, Comes O, et al. The GeneMANIA prediction server: biological network integration for gene prioritization and predicting gene function. Nucleic Acids Res. 2010;38 10.1093/nar/gkq537 PMC2896186 20576703

[19] Livak KJ, Schmittgen TD. Analysis of relative gene expression data using real-time quantitative PCR and the 2(-Delta Delta C(T)) method. Methods. 2001;25(4):402–408. 11846609 10.1006/meth.2001.1262

[20] Ferrandon S, DeVecchio J, Duraes L, et al. CoA Synthase (COASY) mediates radiation resistance via PI3K signaling in rectal cancer. Cancer Res. 2020;80(2):334–346. 31704889 10.1158/0008-5472.CAN-19-1161 PMC7050829

[21] Yang IH, Jung JY, Kim SH, et al. ABT-263 exhibits apoptosis-inducing potential in oral cancer cells by targeting C/EBP-homologous protein. Cell Oncol. 2019;42(3):357–368. 10.1007/s13402-019-00431-5 PMC12994282 30919222

[22] Zhangyuan G, Wang F, Zhang H, et al. VersicanV1 promotes proliferation and metastasis of hepatocellular carcinoma through the activation of EGFR-PI3K-AKT pathway. Oncogene. 2020;39(6):1213–1230. 31605014 10.1038/s41388-019-1052-7

[23] Morshed RA, Young JS, Hervey-Jumper SL, et al. The management of low-grade gliomas in adults. J Neurosurg Sci. 2019;63(4):450–457. 30916536 10.23736/S0390-5616.19.04701-5

[24] Gusyatiner O, Hegi ME. Glioma epigenetics: from subclassification to novel treatment options. Semin Cancer Biol. 2018;51:50–58. 29170066 10.1016/j.semcancer.2017.11.010

[25] Zhu H, Ouyang H, Pan X, et al. Increased ASF1B expression correlates with poor prognosis in patients with gliomas. Front Oncol. 2022;12:912101. 35875094 10.3389/fonc.2022.912101 PMC9298524

[26] Zhang W, Gao Z, Guan M, et al. ASF1B Promotes oncogenesis in lung adenocarcinoma and other cancer types. Front Oncol. 2021;11(731547):731547. 34568067 10.3389/fonc.2021.731547 PMC8459715

[27] Wang W, Xiao L, Pan D, et al. ASF1B enhances migration and invasion of lung cancers cell via regulating the P53-mediated epithelial-mesenchymal transformation (EMT) signalling pathway. Neoplasma. 2022;69(2):361–369. 35103478 10.4149/neo_2021_210818N1181

[28] Misiewicz-Krzeminska I, Sarasquete ME, Quwaider D, et al. Restoration of microRNA-214 expression reduces growth of myeloma cells through positive regulation of P53 and inhibition of DNA replication. Haematologica. 2013;98(4):640–648. 23100276 10.3324/haematol.2012.070011 PMC3659997

[29] Ouyang X, Lv L, Zhao Y, et al. ASF1B serves as a potential therapeutic target by influencing cell cycle and proliferation in hepatocellular carcinoma. Front Oncol. 2021;11(801506):801506. 35087760 10.3389/fonc.2021.801506 PMC8787347

[30] Montes de Oca R, Gurard-Levin ZA, Berger F, et al. The histone chaperone HJURP is a new independent prognostic marker for luminal a breast carcinoma. Mol Oncol. 2015;9(3):657–674. 25497280 10.1016/j.molonc.2014.11.002 PMC5528705

[31] Segura-Bayona S, Stracker TH. The tousled-like kinases regulate genome and epigenome stability: implications in development and disease. Cell Mol Life Sci. 2019;76(19):3827–3841. 31302748 10.1007/s00018-019-03208-z PMC11105529

[32] Klimovskaia IM, Young C, Stromme CB, et al. Tousled-like kinases phosphorylate Asf1 to promote histone supply during DNA replication. Nat Commun. 2014;5:3394. 24598821 10.1038/ncomms4394 PMC3977046

[33] Wang Z, Chen X, Liang Q, et al. Inhibiting of circ-TLK1 inhibits the progression of glioma through down-regulating PANX1 via targeting miR-17-5p. J Mol Histol. 2021;52(5):1007–1020. 34181173 10.1007/s10735-021-09993-x

